# Editorial: Mechanisms and consequences of Aquaporin-4 redistribution in neurological disease

**DOI:** 10.3389/fncel.2023.1143352

**Published:** 2023-02-01

**Authors:** Jenny Szu, Vladimir Parpura, Mahmood Amiry-Moghaddam, Alex J. Smith

**Affiliations:** ^1^Division of Biomedical Sciences, University of California, Riverside, Riverside, CA, United States; ^2^Department of Neurobiology, University of Alabama at Birmingham, Birmingham, AL, United States; ^3^International Translational Neuroscience Research Institute, Zhejiang Chinese Medical University, Hangzhou, China; ^4^Division of Anatomy, Department of Molecular Medicine, University of Oslo, Oslo, Norway; ^5^Department of Ophthalmology, University of California, San Francisco, San Francisco, CA, United States; ^6^Department of Medicine, University of California, San Francisco, San Francisco, CA, United States; ^7^Department of Physiology, University of California, San Francisco, San Francisco, CA, United States

**Keywords:** Aquaporin-4 (AQP4), neurological disease, redistribution, astrocyte, subcellular

Aquaporin-4 (AQP4) is the predominant water channel expressed by astrocytes where it facilitates the bidirectional transport of water across brain fluid interfaces. AQP4 is abundantly expressed at the perivascular endfeet of astrocytes and serves various functions including maintenance of water homeostasis, regulation of cell volume, as well as astrocyte migration. Redistribution of AQP4 has been reported in various neuropathological conditions and targeting subcellular localization of AQP4 may potentially be therapeutically beneficial.

AQP4 isoforms form orthogonal arrays of particles (OAPs) with AQP4-M23 being more dominant in OAPs. The stability of OAPs can impact AQP4 localization and contribute to AQP4 dysfunction. In their review article, Szu and Binder discusses how dysregulation of OAPs due to altered expression of AQP4-M23 can be one mechanism contributing to a pro-epileptigenic effect in the central nervous system (CNS). AQP4-M23 tends to form more sizeable and stable OAPs and are richly expressed at the perivascular endfeet of astrocytes and loss of AQP4-M23 can potentially result in impaired glutamate clearance and enhanced cellular swelling, overall leading to excessive neuronal firing.

The dystrophin-associated protein (DAP) complex is a critical molecule involved in anchoring AQP4 to astrocyte membranes. Redistribution of AQP4 has been attributed to impairments of the DAP complex members, however, it remains unclear as to what signaling pathways are involved in AQP4 subcellular redistribution. Skauli et al. found that canonical bone morphogenetic proteins (BMPs) upregulated AQP4 expression and downregulated α-dystrobrevin (*Dtna*) and dystrophin (*Dmd*), genes that encode for members of the DAP complex. This is the first study to demonstrate AQP4/DAP complex regulation at a pathway level.

Because of its notable role in water transport, AQP4 regulation is critical in brain edema. In their study, Elsherbini et al. investigated the mechanisms driving astrocyte swelling in acute hepatic encephalopathy (HE) in rats. The authors found that rats treated with tioacetamide (TAA) demonstrated increased expression of glial fibrillary acidic protein, tumor necrosis factor α, and AQP4 with a positive correlation with brain water content. Interestingly, a dramatic decrease in expression of potassium inward rectifier channels Kir4.1 was observed suggesting a more significant dysregulation in Kir4.1 in HE-associated brain edema.

Given its proposed role in glymphatic transport, AQP4 expression was evaluated in biopsied specimens in individuals with idiopathic normal pressure hydrocephalus (iNPH). In this study, Eide showed that patients with definite iNPH exhibited AQP4 redistribution away from the endfeet and toward the neuropil with concomitant reduction in dystrophin. The subcellular mislocalization of AQP4 with loss of the anchoring protein resulted in changes in the glia-neuro-vascular interface overall impacting the symptomology of the disease.

Several studies have shown the importance of AQP4 expression in gliomas. However, the role of AQP4 in immune regulation remains to be determined. Interestingly, Lan et al. found that AQP4 expression was strongly correlated with expression of various immune checkpoints and immune cells and negatively correlated with antigen presentation. Different levels of AQP4 expression can also impact tumor malignancy. These findings demonstrate the potential of AQP4 in tumor immunotherapy.

Although alterations in AQP4 expression and subcellular distribution have been widely recognized in various neuropathological states, neither the cellular mechanisms leading to altered AQP4 expression, nor the consequences for disease pathophysiology are well-understood. This Research Topic aims to provide evidence of the cellular and molecular underpinnings involved in AQP4 dysregulation in order to expand our previous understanding of how AQP4 dysfunction impacts the pathophysiology of neurological disorders.

## Author contributions

JS prepared the initial draft of the manuscript. AS, VP, and MA-M revised the manuscript. All authors approved of the final editorial.

